# Disaster‐Related Wound Care: A Scoping Review

**DOI:** 10.1002/nop2.70066

**Published:** 2024-11-21

**Authors:** Qin Shu, Xiang Zhang, Yitong Yuan, Zhifang Li, Wei Ren, Beijing Chen, Fei Xie, Guangyun Hu

**Affiliations:** ^1^ Department of Military Nursing, School of Nursing Army Military Medical University Chongqing China; ^2^ Laboratory of Trauma Care, School of Nursing Army Military Medical University Chongqing China; ^3^ Department of Obstetrics and Gynecology General Hospital of the Northern Theater Command Shenyang Liaoning Province China; ^4^ Department of Neurology General Hospital of Xinjiang Military Region Urumqi Xinjiang China; ^5^ Department of Foreign Language Teaching and Research, College of Basic Medical Sciences Army Military Medical University Chongqing China; ^6^ Department of Basic Nursing, School of Nursing Army Military Medical University Chongqing China

**Keywords:** disaster nursing, disaster relief/rescue, disaster‐related wound care, emergency care, emergency medical team, wound care

## Abstract

**Aim:**

To better understand the research status and demand of society on disaster‐related wound care.

**Design:**

Scoping review.

**Methods:**

A systematic search and screening was conducted in PubMed, CINAHL, Embase, WHO Guidelines, Cochrane Library, ISI Web of Science, MEDLINE and China National Knowledge Infrastructure (CNKI) Database, and 31 articles were selected from 244 articles for critique and synthesis.

**Results:**

Existing disaster‐related wound care research lacks a systematic review. In numerous nations, the technology and administration of disaster‐related wound care for Emergency Medical Teams (EMT) are still nascent, the responsibilities are ambiguous, and there is a shortage of wound professionals. Current critical issues include the following: (1) inadequate strategies for enhanced orderly management of disaster‐related wounds, particularly in an emergency setting and (2) lack of associations and organisations responsible for promoting research and development of catastrophe‐related wound care proliferation strategies in disaster wound care.

**Conclusion:**

There is still a lack of understanding regarding effective organisation and scientific implementation of disaster‐related wound care. More research should be carried out, focusing on the formulation of guidelines and continuous training, so as to promote the standardisation of disaster‐related wound care in the future.

**Abbreviations**
ACSC: ambulatory care sensitive conditions
B‐FAST: belgian first aid and support team
CFR: case fatality ratio
CMO: Chief Medical Officer
DMAT: Disaster Medical Assistance Team
EM‐DAT: International Disaster Database
EMTs: emergency medical teams
FMTs: Foreign Medical Teams
ICN: International Council of Nurses
KDRT: Korean Disaster Relief Team
MMT: Mobile Medical Team
SODs: sudden onset disasters
WHO: World Health Organization
WOC: wound/ostomy and continence

## 1 Introduction

A substantial increase in the number of patients requiring wound care has resulted from the impact of natural disasters on humans. According to World Health Organization (WHO) statistics, 4.4 billion people were injured, lost their residences, were displaced or required emergency assistance between 1998 and 2017 (United Nations 2018). Following a natural disaster, it is possible to find a large number of injured individuals in a short period of time, with the number of casualties rising significantly as time passes (Gill et al. 2021). In addition to being more complicated and severe than typical wounds, disaster‐related wounds are also associated with a variety of post‐disaster infectious diseases, which complicates the treatment of these wounds (Avruscio et al. 2020). To distinguish this form of disaster‐related wound from everyday wounds, the term ‘disaster‐related wounds’ is coined to describe wounds caused or exacerbated by disasters (Ferrari 2015; Osaadon et al. 2018). However, this region has not been studied sufficiently. Consequently, there is a distinct need to systematically assess the current state of disaster‐related wound research and organisation, identify current deficits and determine future efforts' directions.

## 2 Background

### 2.1 Characteristics of Disaster‐Related Wounds

Disasters often can cause a large number of casualties within a few minutes, and fewer trauma cases present to the EMT as time passes (Yitzhak et al. 2018) which require immediate large‐scale medical assistance prior to any comprehensive assessment and wound care (IFRC and WHO 2017). Following the initial large‐scale medical assistance, the next 3–5 days are the peak time period in which the wounded seek emergency medical care (Bartels and VanRooyen 2012). During the third to fifth week after Typhoon Haiyan, for example, the proportion of disaster‐related lesions rose from 18% to 38% (Kim, Moon, and Kim 2018). Importantly, some chronic wounds worsen significantly following a disaster. For example, a retrospective study showed the hospitalisation rates of stage III and IV pressure ulcers in the home care sector increased by 7.7% and 26.4%, respectively, following an earthquake (Fuse and Yokota 2010). This is nearly 10 times greater than the average incidence of pressure ulcer hospitalisations in Japan. Following spinal cord injuries, pressure ulcers have become the second most common disaster‐related injuries (Sato and Ichioka 2012). To help people respond efficiently and provide effective emergency medical services following natural disasters, it is crucial to understand these timelines for injury occurrence and presentation.

The nature of disaster‐related wounds is usually complex and beyond the capacity of local specialised treatments. Earthquake‐related injuries are considered the most challenging type of disaster‐related wound care (Tarif et al. 2016), approximately 30% of earthquake patients have head and neck injuries, 25% have chest, thoracolumbar or spinal cord injuries, 22% have fractures, and 6% have soft tissue contusions, sprains or complicated neurovascular injuries (Bartels and VanRooyen 2012), and typically multiple skin ruptures or deep puncture wounds with tissue compression and destruction. These incisions are frequently contaminated with soil, silt, seawater, sand and detritus, as well as faeces and saliva, and they may be accompanied by fractures, sprains, neurovascular trauma and other life‐threatening injuries (Bartels and VanRooyen 2012). Tsunami‐related wounds are typically superficial lacerations and cuts/scratches, or deeper injuries that damage the fascia, and may be accompanied by fractures, head trauma and other injuries. Wound infection is the most common complication of disaster‐related wounds.

In addition, disasters frequently result in wound infection, which is frequently the result of wound contamination, tissue loss, incomplete or delayed cleansing and debridement, and premature wound closure (Wuthisuthimethawee et al. 2015). For instance, wounds caused by tsunamis and floods are frequently characterised by severe infection, with deep tissue lacerations by pointed objects being the most common cause of wound infection (Lim 2005). The infection rate of typhoon‐related wounds is 44%~59%, mainly due to delayed or inadequate disinfection and foreign body removal (Ferrari 2015; Kim et al. 2016). Severely infected disaster‐related wounds are difficult to treat, especially if combined with septicemia. The incidence of tetanus caused by disaster‐related wounds is also high. Severe complications, such as tetanus, systemic infection and multiple organ failure, caused by mishandling of disaster‐related wounds increased the avoidable hospitalisation rate and led to an increase in disability and death rates (Sasabuchi et al. 2017).

### 2.2 The Crisis of Disaster‐Related Wound Care

Due to the aforementioned characteristics of disaster‐related wounds, patients with such wounds should receive effective wound management as soon as feasible to reduce the risk of complications and promote wound healing (Bartels and VanRooyen 2012; Prevaldi et al. 2016). If it is not treated in a timely manner, not only are complications like wound infection common but also severe cases like tetanus that threaten public health will increase significantly (Finkelstein et al. 2017). However, the management of many disaster‐related wounds requires interprofessional coordination and collaboration, as well as elevated skill requirements for specific wound care practitioners, making disaster‐related wound care a difficult endeavour (Wuthisuthimethawee et al. 2015). Moreover, the burden of disaster medicine in terms of medical costs and multidisciplinary coordination and cooperation is extremely high, which frequently exacerbates the undertreatment of post‐disaster wounds, resulting in an increased rate of wound complications. These indicate the need for wound care capacity enhancements in the aftermath of disasters.

### 2.3 Preparedness of Emergency Medical Teams for Disaster‐Related Wound Care

As an essential component of disaster relief, emergency medical teams play a crucial role in wound care related to disasters. According to the WHO's fact document, EMTs, formerly known as foreign medical teams (FMTs) (Kobi Peleg and Norton 2020), are divided into four distinct categories. All EMTs are responsible for disaster‐related wound care (Yitzhak et al. 2018). Therefore, EMT staff have traditionally focused on trauma and surgery, aiming to respond quickly after disasters or emergencies, and are deployed to affected areas within hours to days to respond to emergency needs. However, disasters often lead to mass occurrence of specific types of wounds, and most EMTs are unfamiliar with the management of complex wounds and may not be able to deliver full‐spectrum disaster‐related wound care. Improving EMT disaster‐related wound care capabilities and bolstering the function of various wound care associations in disaster wound management are essential means for enhancing the quality of disaster‐related wound care.

Studies have shown that possessing sufficient knowledge, skills, facilities and policies to accurately assess disaster‐related wound risks and implementing scientific care procedures to reduce the incidence of disaster‐related wound complications are components of disaster‐related wound care preparations (Kimin et al. 2021; Hughes et al. 2021). However, there is a lack of systematic understanding and discussion of EMTs' current abilities to treat disaster‐related trauma. In addition, there are few studies that investigate the current research on disaster‐related wound care and the future trajectory of this research. Therefore, it is challenging to assist EMTs and emergency nurses in organising and preparing for disaster‐related wound care.

Meanwhile, increasingly, disaster relief practices have shown that the timely establishment of effective wound care teams or organisations with similar functions can correctly manage various disaster‐related wounds and improve the effectiveness of post‐disaster wound care (Wuthisuthimethawee et al. 2015). A few EMTs are aware of the importance of disaster‐related wound care organisations and have established disaster‐related wound care teams. One of the EMTs involved in the Haiti Earthquake established a Post‐Disaster Wound Care Department (Wound Clinic). It is directly managed by the Chief Medical Officer (CMO) of the disaster relief team and is parallel to other departments managed by the CMO, such as Orthopaedics (Surgery), Paediatrics, Internal Medicine and Anesthesiology (Ennis 2010). In Japan's Disaster Medical Assistance Team (DMAT), wound/ostomy and continence (WOC) nurses play a crucial role with disaster‐related pressure ulcer control (Sato and Ichioka 2012). Still, there are many rescue teams, such as the Korean Disaster Relief Team (KDRT) and Canada's Mobile Medical Team (MMT), that treat disaster‐related wounds without wound care certification (Savage et al. 2015; Kim et al. 2010). In China, disaster‐related wound treatment is mostly performed by doctors. Specialised wound care teams led by nurses seldomly participate in on‐site disaster rescue work. More often, nurses deal with leftover wounds and difficult wounds during the post‐disaster recovery period. In short, there is very little research on disaster‐related wound care, with insufficient focus on associations involved in the management of disaster‐related wounds. In addition, there is almost no research interest in the possible deficiencies of disaster‐related wound care management.

To summarise, research on wounds related to disasters is fragmented, encompassing various types of original research such as qualitative and quantitative studies, retrospective analyses, case reports and secondary research. Additionally, expert reviews, research papers focusing on the preparedness, education, response, prevention, and control of first responders, medical institutions, and associations, as well as narrative studies and guidelines, contribute to the existing body of knowledge. In order to achieve this objective, we will undertake a thorough examination and evaluation of the aforementioned genres of literature. The objective of this article is to provide a summary of pertinent information and recent research findings on disaster‐related wound care and to identify potential gaps. This could aid rescuers and wound care specialists in making appropriate judgements and implementing optimal post‐disaster care, as well as improve EMT readiness.

## 3 Method

### 3.1 Research Design

This scoping review seeks to summarise essential information and the findings of recent research regarding disaster‐related wound care in order to assist rescuers and wound care specialists in making appropriate decisions, implementing optimal care following a disaster and enhancing EMT preparedness. We have opted to employ the PAGER (Patterns, Advances, Gaps, Evidence for practice and Research recommendations) framework as a theoretical framework for this scoping review. The purpose of utilising this framework is to identify gaps and advancements in the field of disaster‐related wound care. We believed it would be beneficial to collect information on broader topics that typically do not address well‐defined issues that could predetermine appropriate research designs and do not necessitate a critical evaluation of the quality of the evidence (Caroline Bradbury‐Jones and Isham 2021; Arksey and O'Malley 2005). Although the critical assessment is not obligatory in scoping studies (Munn et al. 2018), the following were nonetheless taken into account throughout the literature review: quantitative studies were assessed using an analytical cross‐sectional approach (Aromataris et al. 2024); narrative studies were evaluated using the Narrative Review Article Quality Assessment Scale (Baethge, Goldbeck‐Wood, and Mertens 2019); and qualitative studies were assessed using the JBI Qualitative Research Assessment Checklist (Lockwood, Munn, and Porritt 2015). By evaluating mixed methods studies, the Mixed Methods Appraisal Tool enhances the study's quality within a restricted scope (Pluye and Hong 2014). That is, mixed methods research (MM) encompasses any combination of approaches that satisfies the following three criteria: (1) at least one qualitative method (QUAL) and one quantitative method (QUAN) are combined; (2) each method is used rigorously; and (3) the data collections, and/or data analyses, and/or results are integrated. Ethical approval was not required.

### 3.2 Search Strategy

**FIGURE 1:**
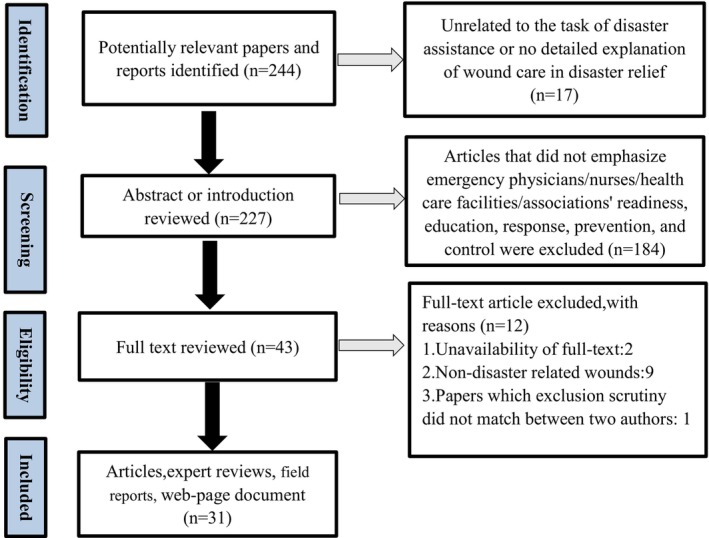
Selection process of papers for review (PRISMA 2009 flow diagram).

The literature search covering January 1, 2000, through January 1, 2022, was conducted in PubMed, WHO Guidelines, Cochrane Library, China National Knowledge Infrastructure (CNKI) Database and Google Scholar. Chinese and English are search languages. The key words ‘natural disaster’, ‘typhoon’, ‘hurricane’, ‘tsunami’, ‘cyclone’, ‘earthquake’, ‘flood’, ‘volcanic eruption’, ‘gale and tornado’, ‘landslides’ were used in combination with the ‘wound’, ‘injury’, ‘crush injury’, ‘open fracture’, ‘burn’, ‘soft‐tissue’, ‘trauma’, ‘lacerations’, ‘cuts’, as well as ‘Organization’, ‘Administration’, ‘emergency medical team’ with the Boolean operation of OR and AND. One of the reviewers searched PubMed for articles meeting the inclusion criteria, and the other searched WHO Guidelines, Cochrane Library, China National Knowledge Infrastructure (CNKI) databases and Google Scholar. Afterwards, all retrieved documents were incorporated into the document management software ‘note‐express’ and all duplicate files were removed. Then, manually check references and citation tracking of included articles to determine other studies that meet the inclusion and exclusion criteria. Full details of the search are shown in Figure 1.

### 3.3 Inclusion and Exclusion Criteria

Inclusion criteria for the papers were as follows:

1. Original research papers (qualitative, quantitative), retrospective studies and case reports and expert reviews about disaster‐related wound care.
2. Research papers, case reports, expert reviews, narrative studies or guidelines about EMTs/healthcare facility/associations preparedness, education, response, prevention and control.

Exclusion criteria were as follows:

1. Papers generally addressing wound care.
2. Papers that did not emphasise emergency physicians/nurses/health care facilities/associations' readiness, education, response, prevention and control.

### 3.4 Screening

The review process consisted of identifying the research question, locating relevant studies, selecting studies, extracting and documenting data, and compiling, summarising, and reporting the results (Arksey and O'Malley 2005). Two reviewers independently reviewed the titles and abstracts of articles and then read the full text of publications that met the inclusion criteria. The PRISMA extension for scoping reviews reporting recommendations (PRISMA‐ScR) (Tricco et al. 2018) were implemented. An article describing the history of disaster relief, as opposed to scientific literature, was eliminated. Thirty‐one articles were selected after a quality appraisal performed by two researchers independently. No relevant guidelines were retrieved.

### 3.5 Data Extraction

The information of the selected articles was converted into data using the following procedure: one reviewer extracted the data for each study using a structured form developed by the researcher; another reviewer confirmed the extracted data. The extracted data included the name of the document's author, author's country, the location and year of the catastrophe, the nature of disaster, the type of study, the medical institutions mentioned and the study's progress. In the exploratory qualitative analysis phase, we first extracted information from the publications regarding EMT team composition and wound care competencies. Subsequently, the research status and prospective research directions of disaster‐related wound care were analysed primarily using the thematic synthesis method of qualitative research within the PAGER framework (Thomas and Harden 2008; Caroline Bradbury‐Jones and Isham 2021). The PAGER framework could aid in the identification of ‘themes (patterns)’, ‘advances’, ‘gaps’, ‘implications for practice’ and ‘implications for research’. Following the PAGER framework will enable us to discuss the ‘contribution and value of disaster‐related wound care research’ in a broader historical context, examine the time period in which these research advancements took place, consider why this is the case, and attempt to influence future research directions.

## 4 Results

### 4.1 Characteristics of the Reviewed Literature

There were 244 research papers being identified from the databases following the searching strategy. After removing any articles that did not comply with the requirements, the final collection consisted of 31 articles, including 30 journal articles and one website page (Table 1).

**TABLE 1:** Characteristics and advances of the reviewed literature.

Author	Author's country	Disaster location (year)	Nature of disaster	Study type	Medical institutions	Advances
Andrew J. Bouland (Bouland et al. 2019)	USA	Bahamas (2019 Hurricane Dorian)	Hurricane	Field report	Team Rubicon (TR)	This article identifies ‘communication, coordination and flexibility of action’ as the three primary coping strategies for addressing the many challenges posed by Hurricane Dorian. The problems associated with disaster‐related wound care have been significantly reduced due to TR's efforts
Sarah Langdon (Langdon et al. 2019)	USA	Montecito, California USA (2018)	Debris Flow	Cross‐sectional study	Three hospitals within Santa Barbara County	The study proposes the ‘debris flow syndrome’ as a unique cluster of symptoms in disaster‐related lesions. Includes extensive abrasions and lacerations of the soft tissues, hypothermia, craniofacial trauma, corneal abrasions, orthopaedic injuries and sludge impaction. Additionally, research reveals that ‘debris flow syndrome’ necessitates highly specialised and coordinated treatment
Maria Moitinho de Almeida (Moitinho de Almeida et al. 2019)	Belgium	Nepal (2015)	Earthquake	Retrospective study	The Tribhuvan University Teaching Hospital (TUTH)	The study clarified that the location of the injury (lower extremities and trunk injuries required longer hospitalisations than head and neck injuries), the need for plastic surgery and amputation and the presence of impact injuries all influenced the duration of hospitalisation for disaster‐related casualties. These results inform disaster‐related wound care decision‐making
Xi Lin (Lin et al. 2017)	China	Nepal (2015)	Earthquake	Review article	National Emergency Medical Rescue Teams of China	The study concludes that medical work in disaster relief should adhere to the principle of ‘specialised treatment combined with multidisciplinary consultation’, particularly in cases of multiple trauma or complex diseases, where multidisciplinary consultation is necessary to assure meticulous treatment
Yitzhak Avraham (Yitzhak et al. 2018)	Israel	Nepal (2015)	Earthquake	Field report	Israeli Emergency Medical Team (IEMT)	The study confirmed that the number of non‐trauma cases increased over time following the disaster and that the hospitalisation rate was approximately 31%. Orthopaedic treatment and wound debridement constituted the majority of disaster‐related wound management
Liu, Y H (刘亚华 et al. 2013)	China	Lushan (2013)	Earthquake	Retrospective study	China International Search & Rescue Team (CISAR)	The study describes the responsibilities of the China National Earthquake Disaster Emergency Rescue Team medical team during the 2013 Sichuan Lushan Earthquake Rescue Mission. These duties include medical security within the team, search and rescue medical cooperation on‐site, medical sites in resettlement areas, patrolling, evacuation of the wounded, psychological intervention, and health and epidemic prevention. The majority of the injuries sustained by the injured were soft tissue injuries and traumas caused by the earthquake. The study also detailed how the earthquake‐ravaged region of Lushan implemented a combination of ‘search and rescue’ and ‘medical rescue’ and accomplished successful rescue efforts
Gerlant van Berlaer (van Berlaer et al. 2019)	Belgium	Philippine (2013 Haiyan)	Typhoon	Cross‐sectional study	The Belgian First Aid and Support Team	The study examined the characteristics of disaster‐related injuries: 59% exhibited signs of infection, 47% required wound care, 33% required pain medication, 29% required antibiotics, 9% of patients required surgery, while 8% need fluid therapy
Yong Won Kim (Kim et al. 2016)	Korea	Philippine (2013 Haiyan)	Typhoon	Retrospective study	Korean Disaster Relief Team (KDRT)	The investigation discovered that 44.4% of disaster‐related wounds were infected. Many post‐disaster wound infections require definitive treatment. Significantly associated with wound infection were foreign body‐contaminated wounds, chronic wounds, time from injury to medical exposure, time to inadequate wound care, and subsequent need for wound management
Savage Erin (Savage et al. 2015)	Canadian	Philippine (2013 Haiyan)	Typhoon	Prospective study	Canadian Armed Forces (CAF) Disaster Assistance Response Team (DART)	This study prospectively created a database for the collection of typhoon Haiyan casualty care data, including the location of mobile medical teams (MMTs), basic patient demographics, the primary causes for patient encounters and any treatment administered. DART has for the first time collected prospective in vitro and clinical data from patients using this prospectively developed database. 28 (11%) of 257 reported trauma patients had disaster‐related injuries, while 214 (83%) had acute post‐disaster injuries. This information assists the Canadian government in determining the optimal moment for missions
Nicholas R Coatsworth (Coatsworth 2014)	Australia	Philippine (2013 Haiyan)	Typhoon	Perspectives	Australian medical assistance team (AUSMAT)	Typhoon‐caused injuries from flying debris at high speeds accounted for a significant proportion, according to the research. As the catastrophe progressed, wound infections increased and type 2 diabetics sustained more severe wounds. The conclusion of the study is that health personnel must be equipped to execute their clinical duties in a disaster setting. It is of the utmost importance to educate and train clinical professional competence in the context of disasters, as well as to cultivate a decent humanitarian attitude and effective work methods
William J Ennis (Ennis 2010)	USA	Haiti (2010)	Earthquake	Empirical Studies	A makeshift tent hospital	The implications of triage in disaster relief and wound care management are discussed in this article. In order to reduce instances of under‐ and over‐triage in the wound clinic, the team suggests coordinating wound care as a distinct clinical entity
Allen Gabriel (Gabriel et al. 2011)	Israel	Haiti (2010)	Earthquake	Special report	Hospital Adventiste d'Haiti of Port‐au‐Prince	This study demonstrates that the adjunctive use of NPWT/reticulated open‐cell foam can shield disaster‐related wounds from external contamination, assist in the removal of wound exudate and infectious material from the wound and stimulate the development of granulation tissue. Consequently, it can promote disaster‐related wound healing and reduce wound complications
Kreiss Yitshak (Kreiss et al. 2010)	Israel	Haiti (2010)	Earthquake	Field report	The Israel Defence Forces Field Hospital	The study posits that proper planning, training, operational diversity and adaptation of treatment guidelines to changing conditions can provide optimal care for the greatest number of patients in a vast number of trauma situations. Thus, it will promote the administration of wounds caused by a disaster
Shannon Doocy (Doocy et al. 2013)	USA	Haiti (2008)	Earthquake	Cross‐sectional study	The Korean Disaster Relief Team (DRT)	The study highlights the inadequate reporting of injuries caused by natural disasters. What requires additional research is as follows: How can the reporting technique be optimised further? How can the report's standardisation and uniformity be enhanced? How to enhance the estimation of casualties in emergency situations, etc. Through these efforts, it serves as a guide for formulating disaster preparedness programmes in various disaster situations
Hoon Kim (Kim et al. 2010)	Korea	Myanmar (2008)	Cyclone	Cross‐sectional study	Korean Disaster Relief Team	The study confirmed that during hurricane‐related calamities, 57.6% of patients presented with acute illness and approximately 5% presented with trauma/injury. The hurricane was directly responsible for 29% of all traumas. The most prevalent diagnostic categories were musculoskeletal issues (21.5%), respiratory abnormalities (15.3%) and digestive system issues (14.6%)
Zhang Jianlin (Zhang et al. 2010)	China	China, Wenchuan (2008)	Earthquake	Cross‐sectional study	A field hospital	This study confirmed the exceptional and irreplaceable function of plastic surgery in field hospitals under severe conditions, particularly in the rescue of victims of powerful earthquakes. It has significantly enhanced the state of disaster‐related wound management and the outcomes for the injured
Chen Enqiang (Chen et al. 2011)	China	China, Wenchuan (2008)	Earthquake	Cross‐sectional study	West China Hospital	The incidence of gas gangrene was approximately 2.41% during the Wenchuan Earthquake. The primary treatment methods included patient isolation, wound exposure and drainage, wound debridement, cleansing or filling with 3% hydrogen peroxide liquid, administration of high doses of antibiotics (primarily Penicillin) to prevent infection, and supportive care to treat symptoms
Qiu Jun (Qiu et al. 2010)	China	China, Wenchuan (2008)	Earthquake	Retrospective study	11 institutions engaged in disaster relief	The study concluded that blunt force trauma, crushing/burial and slipping/falling were the leading causes of injuries resulting from earthquakes. The extremities are the most prevalent lesion site. The most common forms of injuries are fractures and open incisions. Surgical procedures, such as debridement and suturing, closed reduction, and external fixation, are the mainstay of care for these hospitalised earthquake victims
Murat Bozkurt (Bozkurt et al. 2007)	Turkey	Pakistan (2005)	Earthquake	Prospective study	Turkish Red Crescent Field Hospital	Good organisation, teams and apparatus selection are required for a well‐equipped field hospital to provide superior emergency and outpatient care. The lack of orthopaedic and reconstructive surgery specialists on the team has resulted in inadequate capacity for disaster‐related wound care, particularly for victims with exposed fractures and soft tissue defects
Rajpura Asim (Rajpura et al. 2010)	United kingdom	Pakistan (2005)	Earthquake	Field reports	Multi‐disciplinary surgical teams of the United Kingdom	In post‐disaster situations with limited resources, disaster relief teams should have their own teams (including volunteers), equipment and supplies (including disaster‐related wound care supplies) so as not to place additional demands on already limited resources
J.M. Mulvey (Mulvey et al. 2008)	Australia	Kashmir, Pakistan (2005)	Earthquake	Retrospective study	A small military hospital in Forward Kahuta, Pakistan/Mobile Surgical Team (MST)	The study examined the most prevalent forms of Kashmir earthquake‐related injuries, including superficial lacerations (64.9%), fractures (22.2%) and soft tissue contusions/sprains (5.9%). Extremity injuries were the most prevalent (40.1% for upper limbs and 59.9% for lower limbs). At follow‐up, 14.8% had a clinically significant infection necessitating surgical debridement or antibiotic therapy
Kwak Young Ho (Kwak et al. 2006)	Korea	South Asia (2004)	Tsunami	Retrospective study	Disaster medical assistance team	The study confirmed that tsunami‐related injuries accounted for approximately 17.6% of all hospital visits and necessitated extensive surgical treatment and wound care even 2 weeks after the tsunami. Foreign DMAT activities may require efficient triage systems, self‐sufficiency preparations and close cooperation with local authorities
SW Fan (Fan 2006)	Singapore	Banda Aceh (2004)	Earthquake and tsunami	Commentary	The Singapore Armed Forces Medical Team	The study revealed that approximately one‐third of earthquake‐tsunami victims had infected lesions on their extremities and faces. Many patients develop profound necrosis of skin tissue, necessitating multiple wound debridements and dressing changes. Compared to earthquake‐only catastrophes, there were comparatively few cases of severe trauma and fractures
VJ Lee (Lee et al. 2005)	Singapore	Meulaboh, Indonesia (2004)	Earthquake and tsunami	Viewpoint	The Singapore Humanitarian Assistance Support Group	According to the study, 31.8% of emergency department cases and 91.7% of surgical cases were trauma‐related to the tsunami. One week after the calamity, secondary infection and gangrenous wounds reached their apex. In addition, research confirms the significance of preparedness in disaster relief operations, particularly the importance of being able to respond swiftly and unexpectedly to change
David W. Lhowe (Lhowe and Briggs 2004)	Turkey	Not mentioned	not limited	Commentary	International Medical/Surgical Response Teams (IMSRT)	The United States has created a mobile, civilian medical and surgical unit using the past experience of the National Disaster Medical Service and its recent relief efforts following the 1998 African embassy explosions. The International Medical–Surgical Response Team is capable of rapid deployment abroad. It is administered by the Department of Homeland Security and consists of medical and bioengineering professionals from the civilian sector. The organisation's objective is to be able to collaborate with local authorities at mass casualty sites in order to rapidly mobilise the appropriate personnel and equipment in order to assess and stabilise the injured
Sharon E. Mace (Mace et al. 2007)	USA	Not mentioned	Not limited	Retrospective study	Disaster Medical Assistance Teams of American (US‐DMAT)	Despite an increase in ‘man‐made’ disasters such as terrorist attacks in recent years, more than 80% of DMAT team deployments from 1985 to 2002 were to natural disasters, most frequently earthquakes, then hurricanes/tropical cyclones. The role responsibilities are also continuously enriched and enhanced, including the treatment of disaster‐related wound
Ibrahim Arziman (Arziman 2016)	USA	Not mentioned	Not limited	Review	Disaster Assistance Response Team (DART)	Disaster medical teams (or EMTs) consist of physicians, nurses, technicians, administrative logisticians and security personnel, among others. This group must be multidisciplinary, well‐trained, mobile, independent and self‐reliant. During the acute phase (48 to 72 h after the catastrophe), its primary responsibility is to evacuate and rescue affected residents
Government of Canada (2021)	Canadian	Not mentioned	Not limited	Commentary	Mobile Medical Teams (MMTs)	This article describes the mission of the Disaster Assistance Response Team (DART), which was established in 1994 by the Canadian Armed Forces (CAF). Since 1998, Canada has dispatched DART when natural disasters or other calamities strike other nations. It assists when local responders are overburdened and there is nowhere for individuals to go. DART can remain in position for as long as 60 days. It works to stabilise the crisis until long‐term assistance begins to aid the country's recovery
Hisayoshi Kondo (Kondo et al. 2009)	Japan	Not mentioned	not limited	Special report	Japan Disaster Medical Assistance Team (J‐DMAT)	This report introduces the establishment and development of Disaster Medical Assistance Teams (DMATs) in Japan. DMATs are mobile medical teams comprised of highly‐trained paramedics that are designed to meet the requirements of acute emergencies and can be swiftly deployed during the acute phase of a sudden‐onset disaster
Renata Fabia (Fabia et al. 2020)	USA	Not mentioned	Not limited	Review article	Non‐governmental organisations working in burns (B‐NGOs) of Africa	The study identified 27 distinct NGOs operating in burn care in African nations, each with distinctive mandates, capabilities, recruitment methods and disaster response capacities
B. Tarif (Tarif et al. 2016)	Israel	Not mentioned	Not limited	Special report	Forward disaster scout teams (FDSTs)	This study demonstrates that by accumulating and disseminating preliminary information hours after a disaster, forward disaster detection teams (FDSTs) can assist foreign medical teams (FMTs) plan deployment sites and logistics. In addition, the article includes a ‘to‐do’ list for FDSTs so that they can effectively carry out duties that promote FMT readiness

### 4.2 Disaster Type and Author's Country

Twenty‐four of the included articles identified catastrophe categories, including seven natural disasters: hurricane, debris flow, earthquake, typhoon, cyclone and tsunami. Studies on earthquakes are the most prevalent among them (14, 58.33%), followed by typhoons (4, 16.67%) and Tsunamis (3, 12.50%). There are seven documents that do not restrict the categories of disasters but instead focus on the capabilities of disaster rescue teams. The authors of the included papers hailed from 10 different nations. The United States (7, 22.58%) and China (5, 16.13%) are the nations with the greatest number of authors, followed by Israel (4, 12.90%) and Korea (3, 9.68%).

### 4.3 Type and Design of Articles

Fourteen research papers (45.16%) are original; among these, one prospective study (7.16%), six cross‐sectional studies (42.86%) and seven retrospective studies (50.00%) are present. Review or report papers comprised 17 articles (54.84%), of which four were field reports (23.53%), four were commentaries (4.23%), three were special reports (17.65%), three were reviews (17.65%), one was an empirical study (5.88%), and one was a viewpoint (5.88%).

### 4.4 Representative EMTs' Wound Care Capabilities

The disaster rescue teams referenced in this research, comprising EMTs, originated from nine countries: the United States, Canada, China, South Korea, Japan, Turkey, Australia, Africa, and Israel. The majority were from the United States (33.33%), followed by Israel (22.22%). As shown in Table 2, these EMTs are more or less involved in wound care management.

**TABLE 2:** Representative disaster rescue teams and its wound care capabilities.

Team name	Country/organisation	Team composition	Wound care capabilities
Disaster Medical Aid Team (DMAT)	USA	Every DMAT is comprised of a multidisciplinary team of health care professionals, including physicians, nurses, paramedics and EMTs, technicians and others who assist in the mission of the DMAT	Provide medical care for burns, crush injuries, surgeries, etc.
International Medical/Surgical Response Teams (IMSRT)	USA	Physicians with expertise in general surgery, orthopaedics, emergency medicine and anaesthesia compose teams. Teams also consist of registered nurses, surgical technicians and emergency medical technicians with expertise in emergency medicine, trauma and burns	Work in cooperation with local authorities at the mass casualty site to provide rapid assessment and medical stabilisation of injured persons
Team Rubicon (TR)	USA	Typically, TR consists of two mobile treatment units with a physician, a registered nurse and two paramedics on staff	Provide initial outpatient emergency care, basic primary care and basic public health responses to 100 patients/day for a self‐sustained 14‐day period
The Israeli Emergency Medical Team (IEMT)	Israel	There are 124 medical and paramedical staff members on the team, including 44 physicians, 29 nurses and 26 paramedics and medics	Provide definitive wound and basic fracture management Provide complex reconstructive wound and orthopaedic care
Forward disaster scout teams (FDSTs)	Israel	Teams consist of four to six extremely competent individuals, including a logistics officer, a chief medical officer, a team commander, and a communications and information officer	Provide assessment and analysis of injury and wound types Provide assessment of medical facilities and resources
Mobile Medical Teams (MMTs)	Canada	Each MMT typically consists of a minimum of one family physician, one nurse and two medical technicians	Provide primary care including disaster‐related wound care to local residents on a mobile basis
China International Search & Rescue Team (CISAR)	China	The team consists of 39 medical professionals from 25 clinical specialties, such as trauma surgery, orthopaedics, emergency surgery, anaesthesia and dermatology	Provide preventive care and treatment of post‐traumatic complications, functional reconstruction following limb trauma, infection prevention, etc.
Japan Disaster Medical Assistance Team (J‐DMAT)	Japan	The team consists of five to six members, including one or two physicians and two or three nurses. There are plastic surgeons, wound/ostomy and incontinence (WOC) nurses, nutritionists, psychotherapists and one or two logisticians on staff	Treat patients with stage III and IV pressure ulcers Hold a case discussion meeting Instruct other members to deal with stage I and II pressure ulcers Help develop wound care plans for complex wounds
South Korea Disaster Rescue Team (KDRT)	South Korea	Each KDRT team typically consists of seven to seven physicians (including emergency medicine specialists, general surgeons, paediatricians and gynaecologists), four to eight nurses, one to two pharmacists, two to six medical administrators and international rescue personnel	Provide wound assessment, drainage and debridement When patients need surgical treatment and hospitalisation for postoperative management or daily intravenous antibiotics, they are transferred to a nearby hospital
Australian Medical Assistance Team (AUSMAT)	Australia	The team consists of 37 professionals in medicine, nursing, paramedicine and logistics	Provide surgical and trauma care
Turkish Red Crescent Field Hospital	Turkey	In the field hospital, approximately 13 physicians and nine health care assistants rotate every 2–3 weeks	Perform operations, change wound dressings, visit nearby villages to provide help and care
The Burn Non‐governmental organisations (B‐NGOs)	Africa	Contains experts from Asia, Africa, the Middle East, North America, Europe and Canada	Strive to enhance awareness of international burn care needs and to provide information to burn professionals who desire to volunteer their expertise Developing a comprehensive, integrated approach to capacity building and quality improvement for prevention and burn care, specifically in resource poor environments

### 4.5 Observations and Recommendations for Future Studies and Practices

The included literature revealed four patterns, and Table 3 presents the current status, deficiencies, practical evidence and recommendations for future research for these four patterns.

**TABLE 3:** Observations and recommendations for future studies and practices.

Pattern	Advances	Gaps	Evidence for practice	Research recommendations
Concepts and categories of disaster‐related wounds	Existing evidence demonstrates that, on the one hand, disasters can cause new injuries and acute wounds and that, on the other hand, the initial wounds caused by disasters cannot be treated in a timely manner and deteriorate, resulting in a number of complications	Existing research pays insufficient attention to new wounds among various wound‐vulnerable populations (such as immobile patients, diabetic patients, etc.) following natural disasters. Due to a dearth of or insufficient medical resources after the calamity, these patients may not be well‐controlled, resulting in the indirect appearance of new wounds, such as diabetic foot ulcers and pressure sores	Evidence to come to light in future studies	Review the concept or category of ‘disaster‐related wounds’ in a more systematic manner to guide disaster rescue preparations
Characteristics of disaster‐related wounds and the current state of their research	Existing evidence demonstrates that enhanced assessment, timely debridement, prevention and treatment of wound infection, and delayed suturing are essential for disaster‐related wound care. A retrospective survey of disaster relief is useful for summarising and analysing the characteristics of disaster‐related wounds, as well as for summarising wound care experience	Increasing amounts of evidence demonstrate that retrospective studies on disaster‐related wounds lack a systematic design, unified research instruments and prospective observation. Consequently, the disease, characteristics and effects of interventions on disaster‐related wound remain undetermined	Acute wounds caused by disasters are frequently associated with complex injuries and difficult to treat. During disasters, rescue resources are limited, and the likelihood of wounds becoming worse increases	Developing a risk prediction model for disaster‐related wounds may be useful for evaluating the risk of disaster‐related wounds Developing more prospective observational research instruments for disaster‐related wound care may be beneficial for guiding future prospective observational or experimental studies of disaster‐related wound care and for directing data collection It was proposed to create a database of disaster‐related wounds to aid in the formulation and implementation of disaster wound care programmes
Emergency Medical Team Organisations of Disaster‐Related Wound Care	There is some evidence that incorporating disaster‐related wound teams into EMTs or including nurses trained in wound care may enhance the management of disaster‐related wounds	Currently, countries lack academic organisations or specialised management institutions for disaster‐related wound care, and there are few recommendations on how EMT can enhance disaster‐related wound management capacities	Evidence to come to light in future studies	Conducting research on the demand characteristics and demand level analysis of ‘disaster‐related wound care’ in various natural disasters could provide references and suggestions for the future demonstration and promotion of wound care specialist nurses being incorporated into EMT
EMT team training requirements for disaster‐related wound care	There is evidence that EMTs with wound care skills are better able to manage disaster‐related injuries with fewer complications and greater efficiency	Existing research on the knowledge, attitude and skills of EMT members in ‘disaster‐related wound care’ is insufficient, as are training courses and assessment methods	Evidence to come to light in future studies	To improve EMT disaster preparedness, disaster‐related wound care core competencies should be identified, capacity training courses should be built, they should be integrated with relevant ‘disaster preparedness’ training, and a suitable evaluation system should be studied

## 5 Discussion

This scoping review examines the prevalent research on disaster‐related wound care and its voids. Although a few recent studies describing disaster‐related wound care procedures have been published (Wuthisuthimethawee et al. 2015; Kim et al. 2016), the majority of the included studies are retrospective reports based on experience, and their research designs lack rigour. Concerning the effective organisation and empirical implementation of disaster‐related wound care, there is still a paucity of understanding. Below are suggested actions and strategies for the field to facilitate the necessary changes.

### 5.1 Accelerate Research on Disaster‐Related Wound Care

The existing international primary research on post‐disaster wound care strategies is still relatively limited (Wuthisuthimethawee et al. 2015). Moreover, there is even less secondary research on various categories of natural disasters. Due to the limited research, medical conditions, environmental conditions and other factors, the extant disaster‐related wound care strategy is based on experience rather than scientific research (Randolph, Morsch, and Chacko 2019). Our study shows that current research lacks a systematic design, is inadequately in‐depth, lacks prospective trials and is unsystematic. Consequently, the strategies for disaster‐related wound care remain ambiguous and lack evidence‐based guidelines and practices. It is essential to vigorously promote research on disaster‐related wound care, develop guidelines and plans, and set the groundwork for future standardisation of post‐disaster wound care.

### 5.2 Standardise the Competency Requirements for Disaster‐Related Wound Care

The newly proposed concept of disaster‐related wound care more accurately summarises the numerous wound problems that may arise during disasters (Ferrari 2015; Osaadon et al. 2018). However, there is currently no standardised domestic or international description of post‐disaster wound care competencies, and wound care capabilities are not described in the Disaster Nursing Competencies published by the International Council of Nurses (ICN) and the World Health Organization (WHO). In addition, there is a paucity of pertinent research on the competency requirements for wound care personnel in various countries following natural disasters (Sarin et al. 2017, 2019; Hou et al. 2018; Gillani et al. 2021). Recent studies have shown, however, that various disasters require distinct professional competencies (Su et al. 2022). Therefore, pertinent research should be prioritised, local experiences should be compiled as soon as feasible, and meaningful regulations regarding the competency requirements of post‐disaster wound care personnel should be formulated (Su et al. 2022; Zhang, Zhang, and Gong 2021). These actions will aid in standardising the administration of post‐disaster wound care and enhancing its quality.

### 5.3 Disaster Wound Care Requires Ongoing Training

The International Disaster Database (EM‐DAT) classifies disasters into two main categories and nine subcategories: natural disasters and technological disasters. Various categories of disasters necessitate distinct wound management strategies. Rapid technological progress is being made in the treatment of disaster‐related lesions, and more technologies are being implemented (Stadler, Shaban, and Tatham 2016; Chen et al. 2011; Prevaldi et al. 2016). Due to a lack of medical training, however, first responders on the scene of a post‐disaster rescue are frequently unable to recognise the unique characteristics of post‐disaster‐related injuries and, as a result, frequently employ the erroneous rescue methods (Loke, Guo, and Molassiotis 2021). In the case of complex wounds resulting from natural disasters, for example, untrained rescue workers may improperly seal the wounds without performing a thorough debridement, resulting in an increase in tissue infection, a longer wound healing time, an increase in amputations and even systemic sepsis, gangrene or death (Wuthisuthimethawee et al. 2015). New technical requirements for disaster‐related wound care are posed by the global pandemic of emerging infectious diseases (Avruscio et al. 2020). Therefore, there is an urgent need to strengthen training in the necessary skills for disaster‐related wound care, so as to increase the competency of rescue personnel in disaster wound care and enable relevant nursing personnel to articulate their duties in disasters clearly, as well as to continuously improve wound care technology and management. This would provide adequate resources for potential disasters, proficiently share information and identify risks and effectively treat disaster victims' wounds.

### 5.4 Promote and Strengthen Disaster Wound Care Organisations and Management Systems

Establishing disaster‐related wound care organisations before disasters, disaster‐related wound monitoring systems as part of national disaster rescue systems and mechanisms to provide sufficient information to disaster‐related wound care personnel all improve wound management quality (Kearns et al. 2016). Such organisations are useful for organising relationships and constructing reasonable wound care management mechanisms post‐disaster (Born et al. 2007; Choi, Hyun, and Oh 2022; Chen et al. 2020; Saberian et al. 2019; Oldenburger, Baumann, and Banfield 2017). However, the majority of nations lack disaster‐related wound care organisations. In addition, international personnel allocation requirements for disaster‐related wound care are inconsistent. Some nations require certification for wound care therapists, but the vast majority do not. Perhaps including 1–2 wound therapists and others with wound care expertise and experience within the EMT, or a wound care readiness group, and increasing internal rapport through daily training and exercises would be more beneficial for wound care among EMTs. In addition, there should be an increase in the number of wound care practitioners on the Disaster Management and Patient Safety Committee so that they can act swiftly and perform their proper role in the event of a disaster.

## 6 Implications for Nursing & Health Policy

Governments, healthcare policymakers and nursing leaders should promote and strengthen organisations and management systems for disaster‐related wound care, as well as assist medical personnel, particularly EMTs, in receiving professional wound care training. To promote the development of catastrophe wound care, more wound care practitioners should be included in EMT training. Further research should concentrate on the fundamental competencies of disaster‐related wound care for EMTs, and more targeted and systematic disaster‐related wound care courses should be developed.

## 7 Limitations

There are several limitations to this scope review. First, because disasters are often abrupt, catastrophic and exceed local response capabilities, disaster responders frequently lack the time to conduct research on disaster‐related wound care, resulting in publication bias. Second, the majority of the included studies were retrospective, and grey literature, conference abstracts, novels or government documents were also excluded, which may have resulted in bias in the selection of research objects and an emphasis on acute and traumatic wounds. The third limitation is that this survey only considers works first published in English or Chinese. As a result, articles published in other foreign language journals that are related to this review's topic are not included. Future prospective quantitative and combined studies should be conducted to better understand disaster‐related wounds and to aid in decision‐making and policy‐making to enhance future disaster‐related wound care.

## 8 Conclusion

Wound care in the aftermath of a disaster is an emerging and essential topic. Existing studies, which are predominantly retrospective, describe potential methods for managing disaster‐related wound care; however, the relevant research gap has not yet been filled. There is much work to be done, including acting more forward‐looking quantitative and mixed research; more in‐depth discussion on the framework of disaster‐related wound capabilities; more technological innovations in disaster‐related wound management; establishing protocols for carrying out disaster‐related wound care while preventing and controlling infectious diseases (such as COVID‐19), continuously enriching disaster‐related wound care strategies; establish quality training courses; and continue training. This presents both a difficulty and an opportunity. Future disaster care research is likely to place research on disaster‐related wound care at the vanguard, necessitating that more nurses invest in related research and set the groundwork for future evidence‐based practice.

## Author Contributions

All authors have read and agree to the manuscript version submitted for peer review.

## Conflicts of Interest

The authors declare no conflicts of interest.

## Data Availability

The authors have nothing to report.
